# Kernicterus by glucose-6-phosphate dehydrogenase deficiency: a case report and review of the literature

**DOI:** 10.1186/1752-1947-2-146

**Published:** 2008-05-06

**Authors:** Gladys Cossio de Gurrola, Juan José Araúz, Elfilda Durán, Maribel Aguilar-Medina, Rosalío Ramos-Payán, Noemí García-Magallanes, Gerardo Vaca Pacheco, Eliakym Arámbula Meraz

**Affiliations:** 1 Servicio de Genética, Hospital del Niño de Panamá, Panamá; 2 Laboratorio de Biología Molecular, Doctorado en Biotecnología, Facultad de Ciencias Químico Biológicas, Universidad Autónoma de Sinaloa, Culiacán, Sinaloa, México; 3 Centro de Investigación Biomédica de Occidente IMSS, Guadalajara, Jalisco, México

## Abstract

**Introduction:**

Glucose-6-phosphate dehydrogenase deficiency is an X-linked recessive disease that causes acute or chronic hemolytic anemia and potentially leads to severe jaundice in response to oxidative agents. This deficiency is the most common human innate error of metabolism, affecting more than 400 million people worldwide.

**Case presentation:**

Here, we present the first documented case of kernicterus in Panama, in a glucose-6-phosphate dehydrogenase-deficient newborn clothed in naphthalene-impregnated garments, resulting in reduced psychomotor development, neurosensory hypoacousia, absence of speech and poor reflex of the pupil to light.

**Conclusion:**

Mutational analysis revealed the glucose-6-phosphate dehydrogenase Mediterranean polymorphic variant, which explained the development of kernicterus after exposition of naphthalene. As the use of naphthalene in stored clothes is a common practice, glucose-6-phosphate dehydrogenase testing in neonatal screening could prevent severe clinical consequences.

## Introduction

Glucose-6-phosphate dehydrogenase (G6PD) is a housekeeping enzyme that catalyzes the first step in the pentose phosphate pathway, providing reducing power in the form of nicotinamide adenosine dinucleotide phosphate (NADPH). This metabolic pathway is the only source of NADPH in erythrocytes and is therefore the mechanism by which the cell damage caused by oxidative stress is avoided [1,2].

Individuals deficient in G6PD are usually asymptomatic; however, in some cases exposure to chemicals (for example, naphthalene) and drugs (including sulfamides, antipyretics, nitrofurane, primaquine and chloroquine) can induce massive intravascular hemolysis. Among the clinical forms of this enzymatic deficiency are jaundice, acute hemolytic anemia and chronic nonspherocytic hemolytic anemia. A more severe consequence of neonatal hyperbilirubinemia is kernicterus, a neurological syndrome caused by the deposition of bilirubin in the brain tissues, which results in severe consequences and even death [2-4].

G6PD deficiency is an X-linked recessive disease and is the most common human innate error of metabolism, affecting more than 400 million people worldwide [5]. The G6PD gene is highly polymorphic and more than 140 mutations have been described. In the Mediterranean, Middle East, India, China and Southeast Asia, the distribution of multiple alleles account for the total prevalence. There are no reports concerning the prevalence of this enzymatic deficiency in Panama; however, our preliminary studies indicate a high prevalence of G6PD deficiency in this country (unpublished data).

Here we present a kernicterus case in a G6PD-deficient newborn resulting in severe neurological damage.

## Case presentation

### Clinical history

A 4-day-old boy was admitted to the Hospital del Niño in Panama. He was the first child of a healthy young woman from a normal vaginal birth at term. He had an Apgar score of 9 at 1 minute and 9 at 5 minutes, a birth weight of 3.5 kg, body length of 53 cm and a 35 cm cephalic circumference. The newborn was hospitalized when clinical examination revealed 4+ jaundice, hypoactivity, hyporexia and generalized tonic-clonic seizures, requiring management with anticonvulsives, phototherapy and exchange transfusion. A history of use of naphthalene-impregnated clothes was recorded. Analysis of clinical symptoms and laboratory tests diagnosed the proband with kernicterus by G6PD deficiency. Despite clinical management, after 5 years the patient presented with reduced psychomotor development, neurosensory hypoacousia, absence of speech and poor reflex of the pupil to light.

### Laboratory data

**Figure 1:**
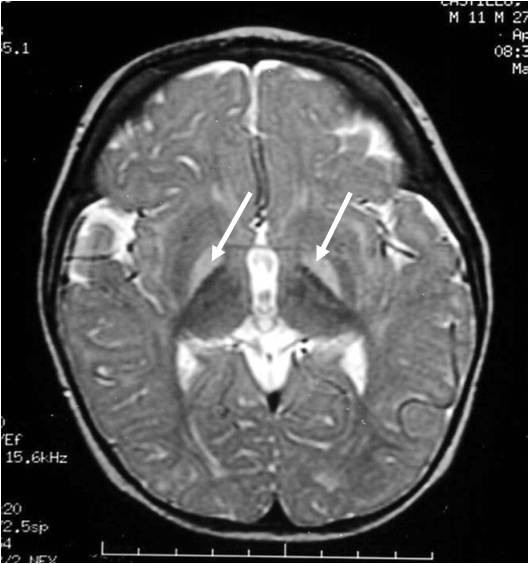
**Magnetic resonance imaging of the head**. Hyperintense basal ganglia lesions on T2-weighted images.

Upon admission the patient presented total bilirubin values of 42.6 mg/dl (41.8 mg/dl from indirect bilirubin), 11.8 g/dl hemoglobin, 8% reticulocytes, O (Rh+) and B (Rh+) blood types for child and mother, respectively, and negative direct Coombs. G6PD testing was reported as deficient, and the quantification of the serum levels of this enzyme was 0.278 U/gHb (normal value range 4.6 to 13.5 U/gHb). Cranial magnetic resonance imaging demonstrated hyperintense basal ganglia lesions on T2-weighted images (Figure 1).

### Mutational analysis

**Figure 2:**
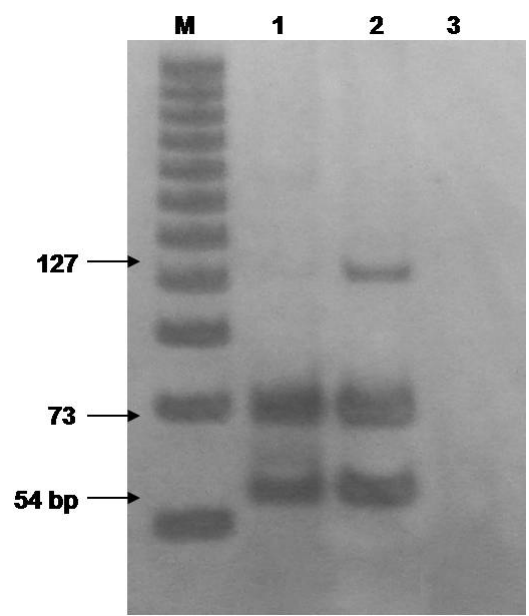
**Polymerase chain reaction and restriction fragment length polymorphism determination**. The G6PD gene was amplified by polymerase chain reaction and digested with *Mbo II*. Lane 1, patient; lane 2, heterozygous control; lane 3, non-template control. M, 25 bp molecular marker.

Determination of the G6PD polymorphic variant was achieved by polymerase chain reaction and restriction fragment length polymorphism (PCR-RFLP) as described elsewhere [6]. Genomic DNA from the patient and one heterozygote G6PD Mediterranean variant control were obtained from heparinized peripheral blood with the salting out method described by Miller et al. [7]. We used 100 ng of DNA to amplify the region flanking nucleotide 563 containing the polymorphism. Amplified products were then digested with the restriction enzyme *Mbo II *and the digestion mix was electrophoresed in 10% polyacrylamide gel. Analysis showed the G6PD Mediterranean polymorphic variant genotype (Figure 2), which explained the G6PD deficiency phenotype.

## Conclusion

G6PD deficiency is an X-linked recessive disease and is the most common human innate error of metabolism, affecting more than 400 million people worldwide [5]. At the Hospital del Niño of Panama, we have observed a high prevalence of G6PD deficiency in our patients; however, there are no reports concerning the prevalence of this enzymatic deficiency in this country.

Here we presented the first documented case of kernicterus in Panama in a G6PD-deficient newborn. The proband presented with hyperbilirubinemia (total bilirubin of 42.6 mg/dl) and was treated with phototherapy and two exchange transfusions. G6PD deficiency testing was positive explaining the symptoms and clinical signs. Mutational analysis demonstrated the G6PD Mediterranean polymorphic variant genotype (Figure 2).

The use of naphthalene-impregnated clothes prior to the episodes of seizure explained the development of severe jaundice that led to kernicterus. As the use of naphthalene in stored clothes is a common practice, so testing for G6PD as part of neonatal screening could prevent this severe clinical consequence.

## Competing interests

The authors declare that they have no competing interests.

## Authors' contributions

GCG provided genetic counseling to the parents. JJA and ED collected the data. MAM, EAM and GVP conducted the data analysis, interpreted experiments and revised the manuscript. MAM, RRP, NGM and EAM performed genetic studies and elaboration, and helped to draft the manuscript. All authors read and approved the final manuscript.

## Consent

Written informed consent was obtained from the patient's next-of-kin for publication of this case report and accompanying images. A copy of the written consent is available for review by the Editor-in-Chief of this journal.
